# New thiophene derivative augments the antitumor activity of γ-irradiation against colorectal cancer in mice via anti-inflammatory and pro-apoptotic pathways

**DOI:** 10.1007/s12672-022-00583-1

**Published:** 2022-11-03

**Authors:** Nermeen M. ElBakary, Sanaa A. Hagag, Mohamed A. Ismail, Wael M. El-Sayed

**Affiliations:** 1 Radiation Biology Department, National Center for Radiation Research and Technology, Egyptian Atomic Energy Authority, Nasr City, Cairo, Egypt; 2 Department of Chemistry, Faculty of Science, Mansoura University, Mansoura, 35516 Egypt; 3 Department of Zoology, Faculty of Science, University of Ain Shams, Abbassia, Cairo, 11566 Egypt

**Keywords:** Apoptosis, COX2, iNOS, Oncogene, PPARγ, p53

## Abstract

**Background:**

Colorectal cancer (CRC) is one of the most common types of cancer worldwide and the second cause of cancer-related deaths. It usually starts as an inflammation that progresses to adenocarcinoma. The goal of the present study was to investigate the antitumor efficacy of a new thiophene derivative against CRC in mice and explore the possible associated molecular pathways. The potential of this thiophene derivative to sensitize the CRC tumor tissue to a low dose of gamma irradiation was also investigated.

**Methods:**

Adult male mice were divided into seven groups; control, group treated with dimethylhydrazine (DMH) for the induction of CRC. The DMH-group was further divided into six groups and treated with either cisplatin, thiophene derivative, γ-irradiation, cisplatin + γ-irradiation, thiophene derivative + γ-irradiation, or left untreated.

**Results:**

DMH induced CRC as evidenced by the macroscopic examination of colon tissues and histopathology, and elevated the activities of cyclooxygenase2 (COX2) and nitric oxide synthase (iNOS). DMH also elevated kirsten rat sarcoma (KRAS) and downregulated the peroxisome proliferator activated receptor (PPARγ) as shown by RT-PCR and Western blotting. DMH exerted anti-apoptotic activity by reducing the expression of phosphorylated p53 and cleaved caspase3 at the gene and protein levels. The flow cytometry analysis showed that DMH elevated the necrosis and reduced the apoptosis compared to the other groups. The colon tissue from DMH-treated mice showed hyperplasia, aberrant crypt foci, loss of cell polarity, typical CRC of grade 4 with lymphocytes and macrophages infiltrating mucosa, muscularis mucosa, and submucosa score 3. Treatment with thiophene derivative or γ-irradiation ameliorated most of these deleterious effects of DMH. The concomitant action of thiophene derivative + γ-irradiation was typified by the better amelioration of tumor incidence and multiplicity, iNOS, PPARγ, p53, caspase 3, and histopathology of colon.

**Conclusion:**

Taken together, the new thiophene derivative is a promising therapeutic candidate for treatment of colorectal cancer in mice. It also sensitizes the CRC tumor to the ionizing radiation through anti-inflammatory and pro-apoptotic pathways.

## Background

Colorectal cancer (CRC) is one of the most common types of cancer worldwide, with 1,931,590 new cases per year estimated in 2020, resulting in significant patient comorbidities and high health-care expenditures [1]. Patients usually need frequent colonoscopies and drastic changes in dietary habits [2]. The development of this cancer starts at the colon mucosa crypts and usually involves inflammation of these crypts. Aberrant crypt foci (ACF) are formed as a result of their altered cellular growth, progressing from microadenoma to adenoma, and finally to adenocarcinoma [3].

The CRC induced by 1,2-dimethylhydrazine (DMH) in rodents is the most common model because this model retains many similarities to human CRC [4]. DMH is metabolized in the liver forming active intermediates such as methylazoxy methanol (MAM) and azoxymethane (AMO). These intermediates are transported with the bile reaching the colon epithelium. The further decay of MAM results in the formation of methyl diazonium ions. These ions cause DNA methylation which is the main inducer of colon polyps and malignant tumors [5].

Given the high prevalence and mortality of colorectal cancer patients, innovative therapeutic agents are desperately needed to improve treatment outcomes in clinics. Depending on the stage, CRC could be treated with surgery only, or with surgery along with chemotherapy, radiotherapy, or sometimes combined chemoradiation. Chemotherapy, which employs cytotoxic substances to kill cancer cells, is still a viable treatment option for CRC patients. Chemotherapy is performed before or after surgery, depending on the stage of colorectal cancer. Cisplatin (CP), 5-fluorouracil, oxaliplatin, leucovorin, irinotecan, and capecitabine are some of the most regularly used anticancer drugs for CRC [6]. However, because of the low response rate and development of drug resistance, safe and effective chemotherapeutic drugs are still in great demand for treating CRC patients. CP causes DNA intercalation preventing the cell growth and metastasis [7]. However, the use of CP is usually constrained because of its nephrotoxicity [8]. CP is accumulated in renal tubular cells causing oxidative stress and inflammatory cytokines burst [9].

**Fig. 1:**
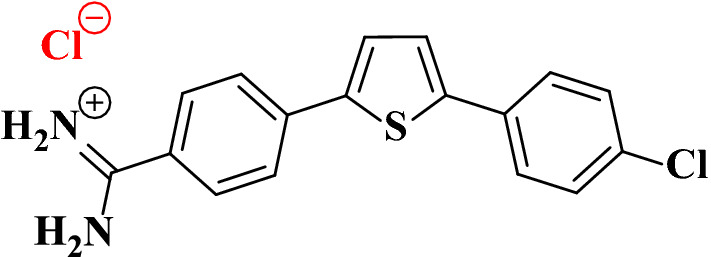
2-(4-Amidinophenyl)-5-(4-chlorophenyl) thiophene hydrochloride salt [13]

Many anticancer drugs have been discovered and developed. Several cationic aromatic compounds, particularly thiophene-based molecules, have recently been found to attach to AT-rich locations in the DNA minor groove and exert anticancer activity [10–14]. These molecules bind to DNA and inhibit one or more of the enzymes required for replication of DNA and thereby, hindering proliferation [13, 15, 16]. In the current study, the efficacy of 2-(4-amidinophenyl)-5-(4-chlorophenyl) thiophene hydrochloride salt (from now on referred to as thiophene derivative, Fig. 1) against CRC was compared to cisplatin (CP). This thiophene derivative (referred to as 4i compound in our previous publication [13]) has a potent antiproliferative activity measured against 60 cancer cell lines at the National Cancer Institute with a median growth inhibition (GI_50_) of 0.2 µM. The GI_50_ values measured against seven colon cancer cell lines; COLO 205, HCC-2998, HCT-116, HCT-15, HT29, KM12, and SW-620 were 0.17 to 0.20 µM with selectivity index of 654 [13]. This means that this compound is very safe to the normal cells. In addition, it caused a 98% inhibition of tyrosine kinase activity at 13 nM [13] presenting itself as a potential antitumor candidate.

Low-dose γ irradiation has been established as a promising supplementary technique for improving the immune adaptive response, and its application in cancer therapy strategies has shown a number of noteworthy outcomes [17]. Radiation hormesis theory proposes that modest doses of ionizing radiation are advantageous by promoting the activation of disease-fighting repair mechanisms that are not active in the absence of ionizing radiation. A low dose of ionizing radiation produces reactive oxygen species that are sufficient to excite the defensive systems and cause observable health benefits. It is usually is in the range of 0.01–0.50 Gy [18]. As a result, the radiation adaptive response directs DNA repair which appears to be the key of the potential hormetic impact [19].

Therefore, the present study was launched to evaluate the antitumor activity and the possible associated molecular pathways of a new thiophene derivative and low dose gamma irradiation. In addition, the possible augmentation of the concomitant treatment with the thiophene derivative and low dose gamma irradiation was also investigated.

## Material and methods

### Animals

Adult male Swiss albino mice (4-month old) weighing 25–30 g were obtained from the National Center for Radiation Research and Technology's (NCRRT). Mice were acclimatized for laboratory conditions for 1 week, and kept in temperature (25 ± 2 ºC) and humidity (60 ± 5%) controlled rooms. During this time, the animals were fed on a commercial standard pellet diet and had free access to water.

### Ethics statement

The study was conducted in accordance with the recommendations in the Guide for the Use and Care of Laboratory Animals of the National Institute of Health (NIH no. 85:23, revised 1996) and in compliance with the regulations of Ethical Committee (REC) of the NCRRT, Atomic Energy Authority, Cairo, Egypt (Approval No: 30A/21). REC has approved this research protocol, following the 3Rs principles for animal experimentation (Replace, Reduce, and Refine) and is organized and operated according to the CIOMS and ICLAS International Guiding Principles for Biomedical Research Involving Animals 2012. The Ethics committee allows tumor burden of 2 cm or about 20% of the body weight in mice. The maximum tumor burden recorded in the current study did not exceed 20% of the body weight.

### Drugs and chemicals

Dimethylhydrazine (DMH), cisplatin, and urethane were obtained from Sigma Aldrich (Sigma-Aldrich, St. Louis, MD, USA). The thiophene derivative was synthesized and fully characterized as described in our previous publication where it was designated as 4i [13].

### Radiation facility and irradiation procedures

Mice were exposed to fractionated dose gamma irradiation of three fractions; 0.25 Gy/week up to a total of 0.75 Gy starting after the induction of colon cancer and thiophene treatment, at the NCRRT utilizing a Canadian Gamma-cell-40 (Cs137) biological irradiator (Canada Ltd. in Ottawa, Ontario, Canada). The animals were placed in a plastic sample tray with a lid and supports for use in the sample cavity. According to the Protection and Dosimetery Department's requirements, the unit contains ventilation holes that align with ventilation sections through the main shield to give a means for uniform irradiation of small animals at a dosage rate of 0.403 Gy/min at the time of experiment.

### Induction of colorectal cancer

1,2-Dimethyl hydrazine (DMH) was dissolved in 1 mM EDTA just prior to use and the pH was adjusted to 6.5 with 1 mM sodium bicarbonate to ensure the stability of the chemical. Animals were given a subcutaneous (s.c) injection of DMH (20 mg/kg body weight) once a week for nine weeks [20, 21]. In addition, dextran sodium sulphate was given at the concentration of 2% in drinking water (2 g/ 100 ml water) for one week starting at the second week of DMH administration. It acts as a promoter in the formation of CRC [22].

### Experimental design

A total number of 130 male albino mice were randomly allocated into seven groups as follows; Group 1 (C): Normal control mice (10 mice received intraperitoneal (i.p.) injection of isotonic saline twice a week over a period of 30 weeks). Group 2 (DMH): 20 mice received DMH (20 mg/kg, s.c. once a week for 9 weeks. Group 3 (DMH + Cis): 20 mice received DMH then cisplatin (5 equal doses of 2.5 mg/kg i.p. over a period of 21 weeks. Group 4 (DMH + T): 20 mice received DMH then the thiophene derivative (2 mg/kg, i.p., 3 doses/week for 21 weeks based on a preliminary study calculating the LD_50_). Group 5 (DMH + R): 20 mice received DMH then whole body was exposed to a fractionated low dose gamma radiation (0.25 Gy) three times over 21 day with a total of 0.75 Gy. Group 6 (DMH + Cis + R): 20 mice received DMH then cisplatin and then exposed to fractionated low dose gamma radiation. Group 7 (DMH + T + R): 20 mice received DMH then thiophene derivative and then exposed to fractionated low dose gamma radiation. At the end of experimental period (30 weeks), animals were scarified using urethane for anesthesia. Colon tissues were collected for biochemical analyses and molecular investigations. Colon sections were prepared for histopathological study. At the end of the experiment, the tumor incidence, multiplicity, and numbers were all calculated and recorded. Tumor incidence = (number of tumor bearing mice/total number of mice) × 100. Multiplicity = total number of tumors / total number of mice bearing tumors.

### Quantification of inflammatory cytokines

Colonic inducible nitric oxide synthase (iNOS) and cyclooxygenase (COX2) activities were determined using mouse specific antibody ELISA kit (abcam, USA; CAT # ab253219 and ab210574, respectively) according to the manufacturer’s instructions.

### Estimation of cleaved caspase-3 and phosphorylated p53 levels

Colonic tissue content of cleaved caspase was determined utilizing mouse active caspase-3 (A-CASP3) ELISA kit (MyBiosource Inc., San Diego, CA, USA; CAT # MBS7210856) according to the manufacturer’s instructions. Phosphorylated p53 was detected using mouse specific antibody ELISA kit (RayBiotech Life, Inc. Code # PEL-P53-S15-1), according to the manufacturer’s instructions.

### Quantitative real-time PCR

RNA was extracted from the colon tissue homogenate using the RNeasy plus mini kit (Qiagen, Venlo, The Netherlands), according to the manufacturer’s instructions. mRNA was reverse transcribed by First Strand cDNA Synthesis Kit (Thermo Scientific co., USA) according to manufacturer’s instructions. The qRT-PCR was performed using RNA-direct SYBR Green Real Time PCR master mix (Invitrogen™) on Mx3000P qPCR system (Agilent Technologies, California, USA). The sequences of the forward and reverse primers for murine *p53, caspase-3,* peroxisome proliferator activated receptor (*PPARγ)*, and kirsten rat sarcoma *(KRAS)* genes were quoted from previous studies [23, 24] as shown in Table 1. The CT values were obtained and normalized to the house keeping gene (*GAPDH*). Fold changes were calculated using 2^−ΔΔCT^ method.

**Table 1:** Primer sequences of investigated genes

Primer	Sequence
*GAPDH*	5′-CAGGAGCGAGACCCCACTAACAT-3′ 5′-GTCAGATCCACGACGGACACATT-3′
*p53*	5′-GGCAACTATGGCTTCCACCT-3’ 5′-AACTGCACAGGGCACGTCTT- 3′
*Caspase-3*	5’-CAAGTCAGTGGACTCTGGGA-3′ 5′-CGAGATGACATTCCAGTGCT-3’
*PPARγ*	5′-GTCCAACAGGAGCATGTGCA-3’ 5′-CCAGCGGTCAATCATACCCA-3’
*KRAS*	5′-GGCCAGGAGTGCATTAAGAC-3’ 5′-GCACGTCAGATAGTCTCCAAA-3′

### Western blot analysis

Colonic PPARγ and KRAS were analyzed by Western immunoblotting. Colon tissue was homogenized using radioimmunoprecipitation assay (RIPA) lysis buffer (Sigma–Aldrich, St. Louis, MD, USA) and protein levels were determined with a BCA protein assay kit (Thermo Fisher Scientific). After extracting the protein, an aliquot of 30 mg protein was denatured, and each sample was loaded onto 8% sodium dodecyl sulphate polyacrylamide gel electrophoresis (SDS-PAGE) and transferred to a nitrocellulose membrane (Amersham Bioscience, Piscataway, New Jersey, USA) using a semidry transfer apparatus (Bio- Rad, Hercules, CA, USA). To ensure homogenous protein transfer, the nitrocellulose membrane was dyed in a Ponceau solution (Sigma–Aldrich, St. Louis, MO, USA) for 30 s. At 4 °C overnight, the membranes were blocked with 5% non-fat milk blocking buffer containing 10 mM Tris–HCl (pH 7.4), 150 mM NaCl, and Tris-buffered saline with 0.05% Tween-20 (TBST). The membranes were then washed with TBST and incubated overnight on a roller shaker at 4 °C with a 1:1000 dilution of anti- PPARγ, and anti-KRAS antibodies. After washing, the filter was probed with the appropriate horseradish peroxidase (HRP)-conjugated goat anti-mouse immunoglobulin (Amersham, Life Science Inc., USA). Detection of chemiluminescence was performed with the Amersham detection kit following the instructions of the manufacturer and then exposed to X-ray film. Quantification of studied protein amount was performed by densitometric analysis of the autoradiograms using a scanning laser densitometer (Biomed Instrument Inc., USA). Finally, the results were normalized against β-actin protein [25].

### Annexin V detection of apoptosis by flow cytometry analysis

For flow cytometry, colonic tissues were dissected and minced to small clumps, followed by enzymatic dissociation with 0.5 mg collagenase IV (Sigma) in PBS for 25 min at 37° C on a shaker, and centrifuged at 500xg for 10 min. The supernatant containing debris was discarded, and the pelleted cells were washed twice with PBS and filtered through a 100 µm cell strainer to obtain a single-cell suspension. cells were washed with cell staining buffer, centrifuged at 400 xg for 5 min at 4 °C, and the supernatant was discarded. The pellet was then resuspended in cell staining buffer, a cell count and viability analysis were performed using trypan blue and the bright line haemocytometer where the suspension was adjusted to a concentration of 1 × 10^6 ^cells/mL. Phosphatidylserine exposure on the outer leaflet of the plasma membrane was detected using the Annexin V-FITC/PI Apoptosis Detection Kit (BD pharmingin TM, BD Biosciences Co., USA; Number # 51-66121E), where cells were then resuspended in 1X Binding Buffer at a concentration of 1 × 10^6^ cells/ml. To a 5 ml culture tube, 5 µl of FITC Annexin V and 5 µl PI were added to 100 µl of the solution (1 × 10^5^ cells). The cells were gently vortexed and incubated for 15 min at 25 °C in the dark. Finally, 400 µl of 1X Binding Buffer was added to each tube and 10,000 cells were analyzed by flow cytometry within an hour on a FACSC-LSR (Becton and Dickinson Company) equipped with Cell Quest software. Three specimens were analyzed from each group.

### Histopathological examination

Tissue specimens were collected from colon fixed in formalin 10% and trimmed off, washed, and dehydrated in ascending concentrations of ethanol. The specimens were then cleared in xylene, embedded in paraffin blocks and sectioned at 5 µm thick. The sections were deparaffinized using xylol and stained with hematoxylin and eosin (H&E) for histopathological examination [26]. The following morphological characteristics were assessed using the standard definitions of the World Health Organization's and the American Joint Committee on Cancer. The tumor cell architectural complexity (arbitrarily graded 1–5; 1 = simple or well differentiated, 2 = some complexity with glands containing apparent secondary structure, 3 = more complexity with glands containing tertiary structures, 4 = complex glands with some areas of solid growth, 5 = solid growth; tumor infiltrating lymphocytes, referring to lymphocytes within tumor cells and tumor nests, exclusive of stromal lymphocytes). Based on the number of lymphocytes, grade 5 has a score range of 0 to 3 (0 = none, 1 = less than 10 per medium power field, 2 = 10–20 per medium power field, 3 = more than 20 per medium power field) [27].

### Statistical analysis

The distribution of data was examined using the Kolmogorov–Smirnov test. Statistical analysis was carried out using ANOVA by GraphPad Prism® version 8.00 (San Diego, CA, USA) software followed by Tukey-HSD test for multiple comparisons. The significant differences were considered statistically at p < 0.05. Data were presented as mean ± SEM.

## Results

### Macroscopic examination of colon, mortality, tumor incidence and multiplicity

**Fig. 2:**
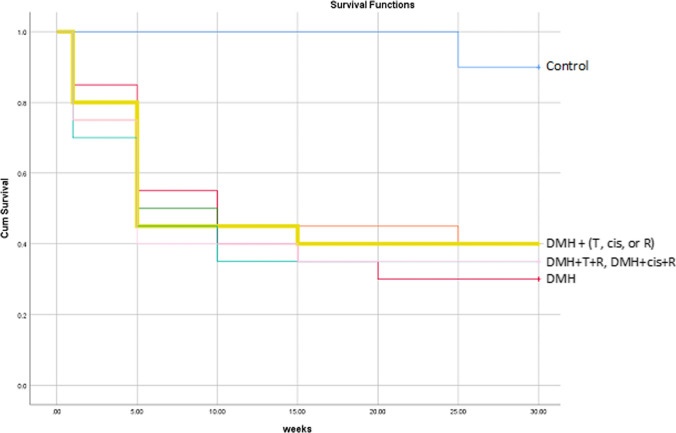
The Kaplan Meier analysis of survival

**Fig. 3:**
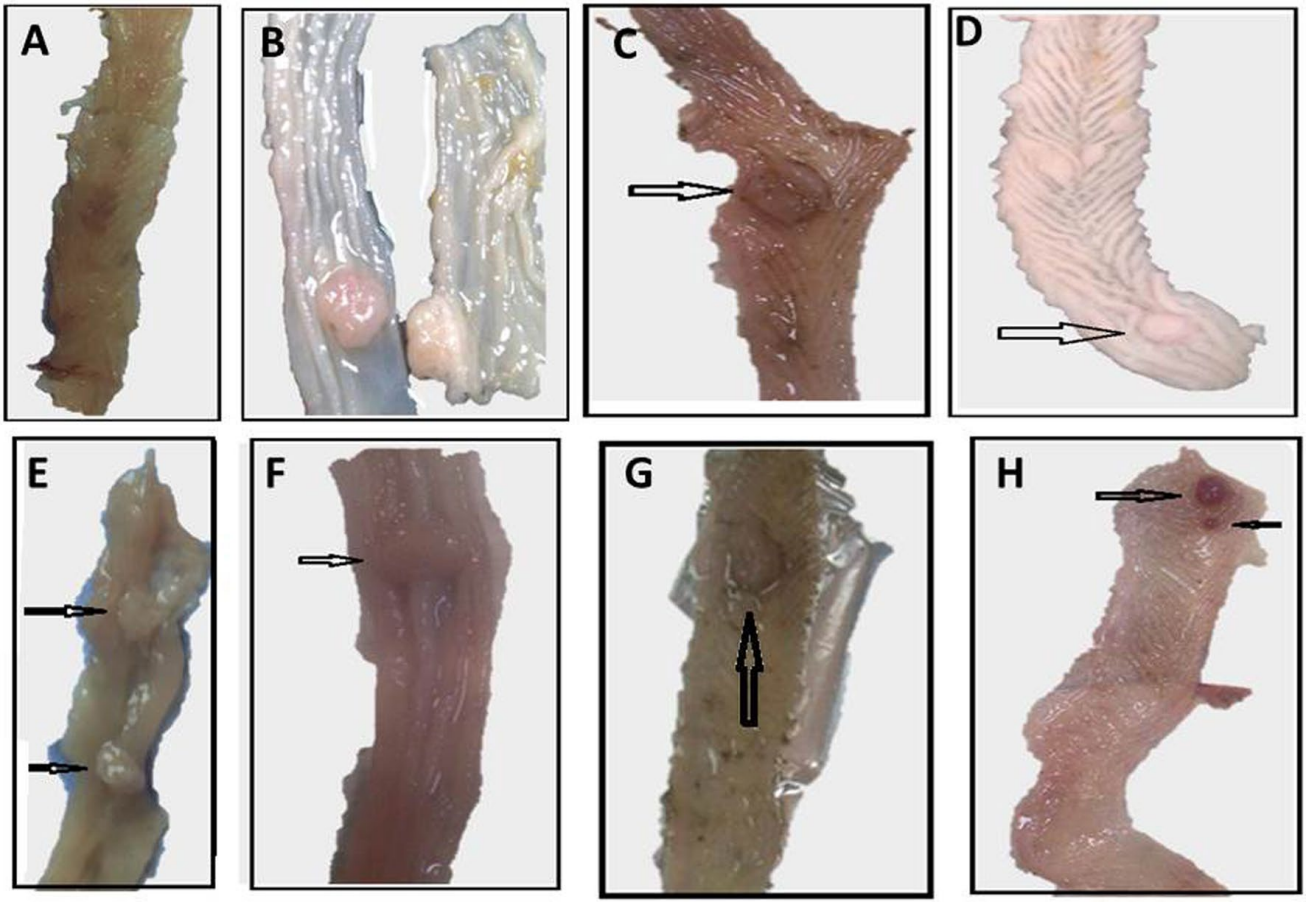
Macroscopic examination of colorectal tissue from **A** Control, **B** DMH, **C** DMH + Cis, **D**, **E** DMH + T, **F** DMH + R, **G** DMH + Cis + R, **H** DMH + T + R groups, The arrows refer to polyps of colorectal cancer

There were no spontaneous tumors in the control group after 30 weeks. DMH resulted in a 70% mortality and this percentage was reduced to 60% in the monotherapy groups, and to 65% in the groups treated with thiophene derivative + irradiation and cisplatin + irradiation. As shown in the Kaplan Meier analysis, most of the mortalities (45–60%) occurred during the administration of DMH (9 weeks). Over the course of 21 weeks after the administration of DMH, all the treated groups lost one or two animals and had a mortality rate at 5–10% (Fig. 2). DMH resulted in 100% tumor incidence. Cisplatin and irradiation equally reduced the incidence to 50%. Thiophene derivative alone reduced the incidence to 37.5%. Concomitant treatment with thiophene derivative and radiation resulted in the best reported incidence (28.5%). This last group also showed the best therapeutic effect on the tumor multiplicity. Monotherapy with thiophene derivative, radiation, or cisplatin reduced the multiplicity by ~ 35–46%. The combined treatment with thiophene derivative + irradiation and cisplatin + irradiation reduced the multiplicity by ~ 77% and 70%, respectively (Table 2, Fig. 3).

**Table 2:** Effect of thiophene derivative and radiation on the mortality rate, and tumor incidence, multiplicity, and count

Groups	Mortality (%)	No. of animals examined	No. of tumors bearing mice	Tumor incidence%	Total no. of tumors	Tumor multiplicity
Control	10	9	0	0	0	0
DMH	70	6	6	100	26	4.3
DMH+T	60	8	3	37.5	7	2.3
DMH+Cis	60	8	4	50	11	2.8
DMH+R	60	8	4	50	10	2.5
DMH+T+R	65	7	2	28.5	2	1.0
DMH+Cis+R	65	7	3	42.8	4	1.3

Tumor incidence% = (number of tumor bearing mice/total number of mice)*100. Multiplicity = total number of tumors/total number of mice bearing tumors. Abbreviations; DMH: dimethylhydrazine, T: thiophene derivative, Cis: cisplatin, R: radiation

### Anti-inflammatory effects of different treatments

**Fig. 4:**
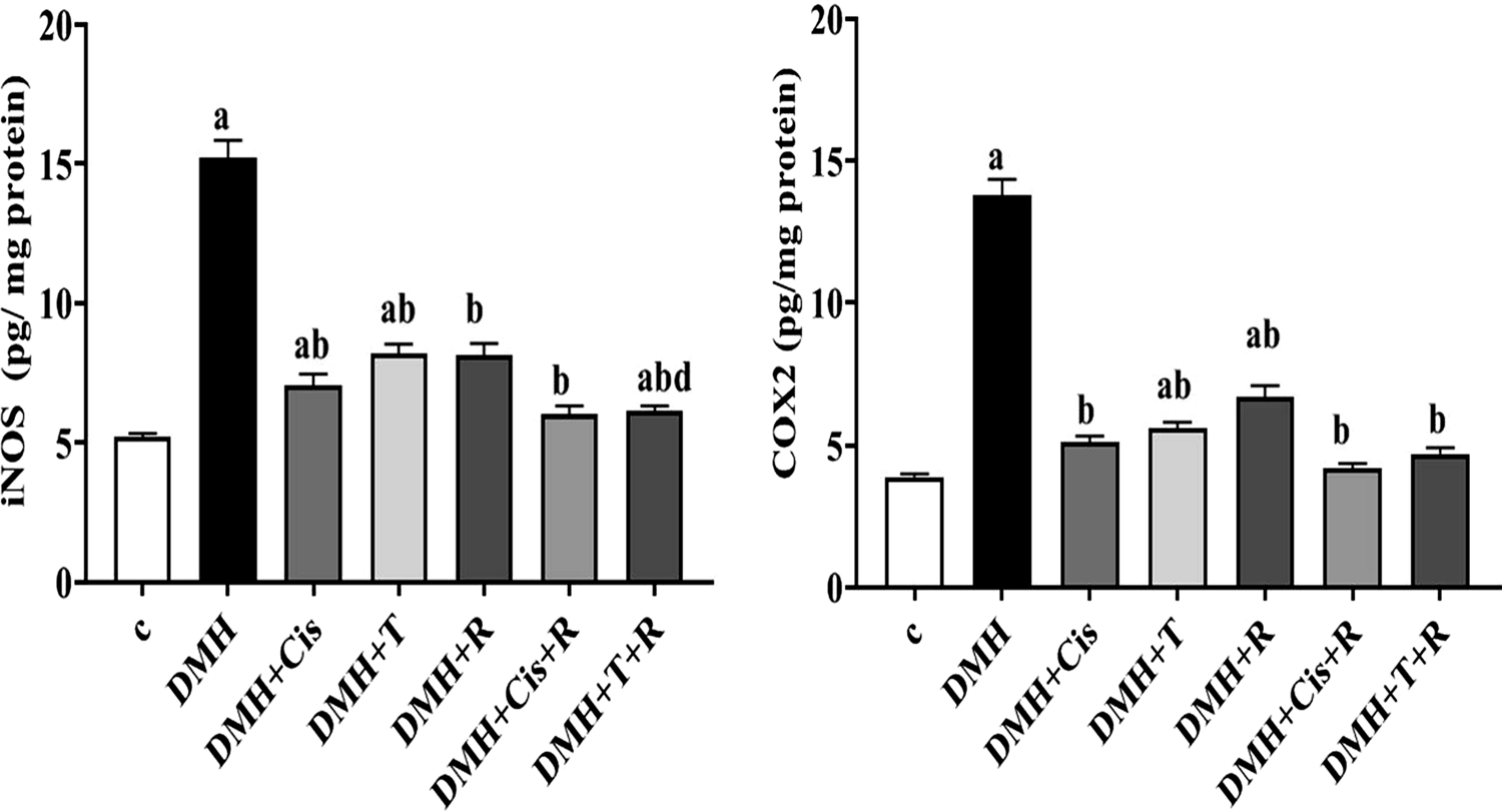
Anti-inflammatory effect of thiophene derivative and low dose γ-irradiation in colorectal tumors in mice. C: control, DMH: dimethyl hydrazine, Cis: cisplatin, T: thiophene derivative, R: radiation. Values are the mean ± SEM (n = 6). ^a^Significant change compared to the control group. ^b^Significant change compared to DMH group. ^d^Significant change compared to DMH + T

The higher activities of iNOS and COX2 in colon homogenates of mice treated with DMH indicated the induction of inflammation compared with healthy control mice. Treating the DMH-mice with cisplatin, thiophene derivative, or radiation resulted in significant (p < 0.05) reductions in the iNOS and COX2 activities compared with the mice treated with DMH alone. Interestingly, exposure of mice treated with DMH to low dose γ-irradiation in combination with thiophene derivative or cisplatin produced a further suppression in iNOS and COX2 activities (Fig. 4). However, this further suppression achieved a statistical significance only in the group treated with the combined thiophene derivative and radiation and only in the iNOS activity. Adding radiation to the therapeutic regimens along with thiophene derivative or cisplatin brought the COX2 activity to the normal control levels.

### Effect of thiophene derivative and γ-irradiation on *KRAS *and *PPARγ* expression

**Fig. 5:**
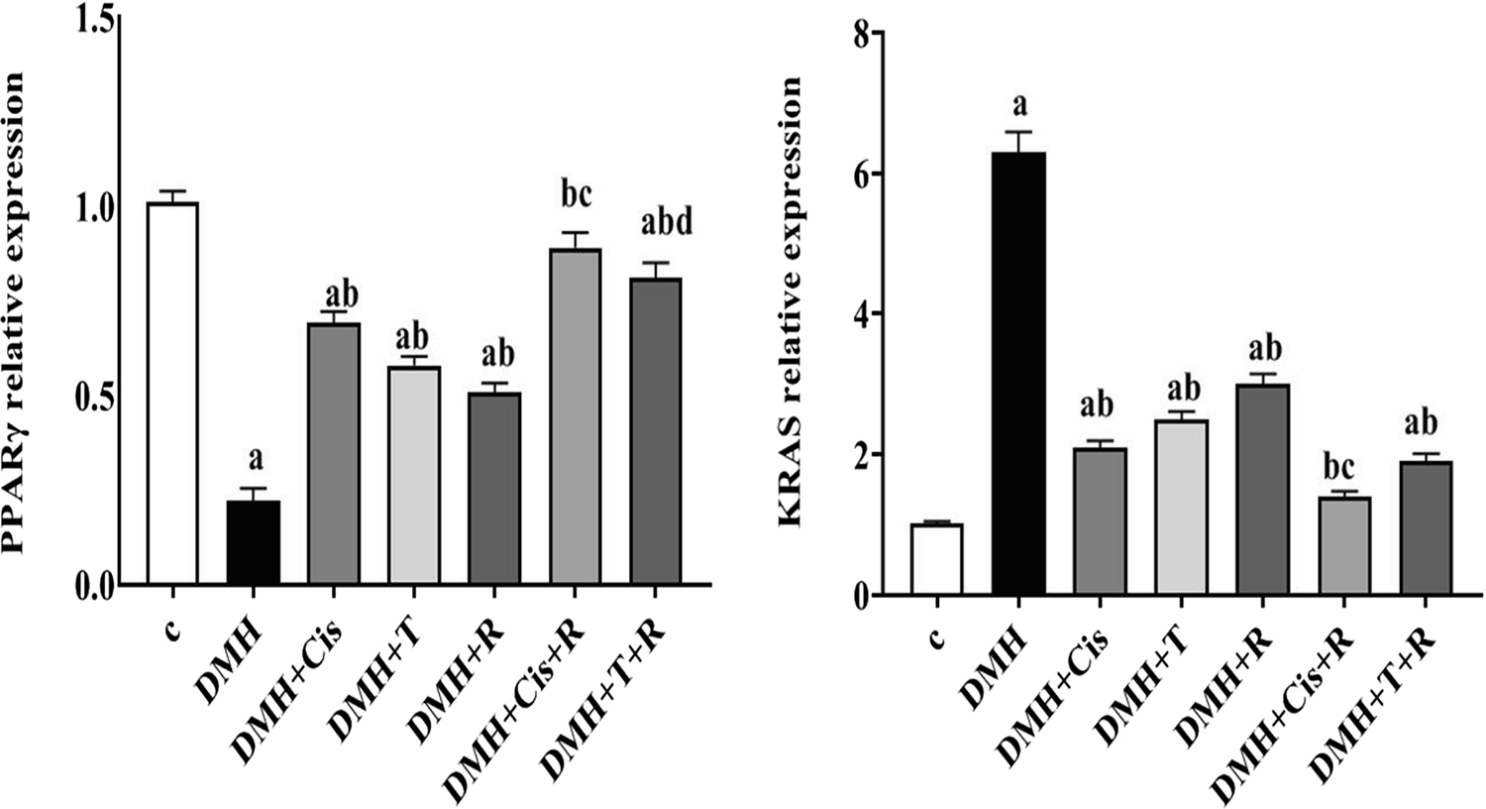
Effect of concomitant treatment with thiophene derivative and low dose γ-irradiation on *PPARγ* and *KRAS* expression. C: control, DMH: dimethyl hydrazine, Cis: cisplatin, T: thiophene derivative, R: radiation. Values are the mean ± SEM (n = 6). ^a^Significant change compared to the control group. ^b^Significant change compared to DMH group. ^C^Significant change compared to DMH + Cis. ^d^Significant change compared to DMH + T

RT-PCR analysis showed a pronounced increase (p < 0.05) in *KRAS* mRNA level and a significant decrease (p < 0.05) in *PPARγ* transcript level in DMH-treated animals as compared to the healthy control group. In DMH + T, DMH + cis, or DMH + R-treated animals, *PPARγ* gene expression was partially increased and *KRAS* gene expression was significantly reduced compared to the DMH group. Moreover, combined treatment with thiophene derivative or cisplatin and low dose γ-irradiation restored *PPARγ* and *KRAS* to levels comparable with those of the normal healthy controls (Fig. 5).

### Effect of thiophene derivative and low dose γ-irradiation on KRAS and PPARγ protein levels

**Fig. 6:**
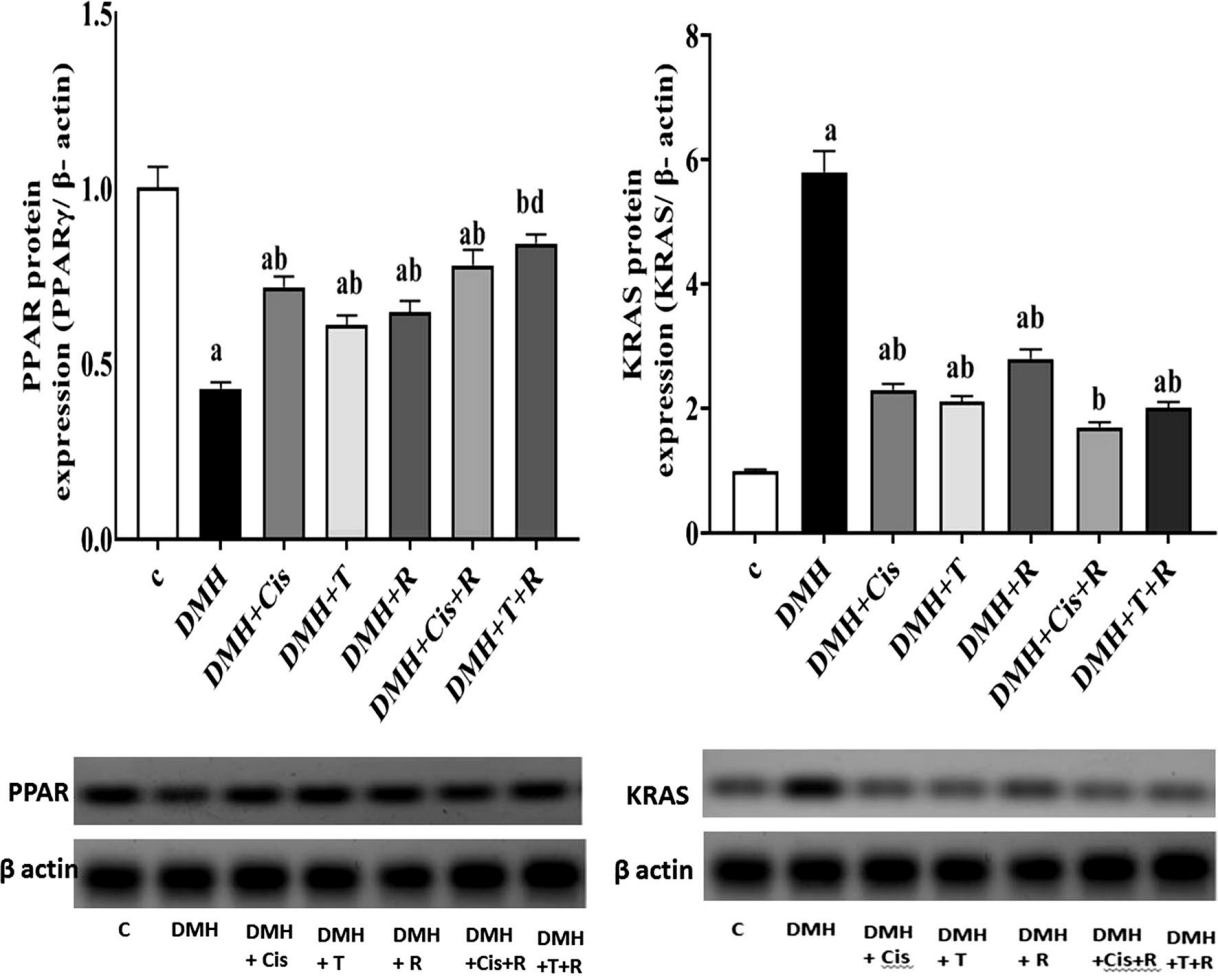
Effect of thiophene derivative and low dose γ-irradiation on PPARγ and KRAS protein levels. C: control, DMH: dimethyl hydrazine, Cis: cisplatin, T: thiophene derivative, R: radiation. Values are the mean ± SEM (n = 3). ^a^Significant change compared to the control group. ^b^Significant change compared to DMH group. ^d^Significant change compared to DMH + T

As compared to healthy control group, there was a noteworthy over-expression of KRAS (p < 0.05) and a remarkable down regulation in PPARγ protein expression in DMH-treated animals. Treatment of DMH-mice with thiophene derivative, cisplatin, or radiation was associated with a significant amelioration in PPARγ and KRAS protein expression. Exposure of tumor bearing mice to γ-irradiation in combination with thiophene derivative or cisplatin significantly augmented the effect of thiophene derivative on PPARγ (Fig. 6).

### Effect of thiophene derivative and low dose γ-irradiation on *p53* and *caspase-3* expression

**Fig. 7:**
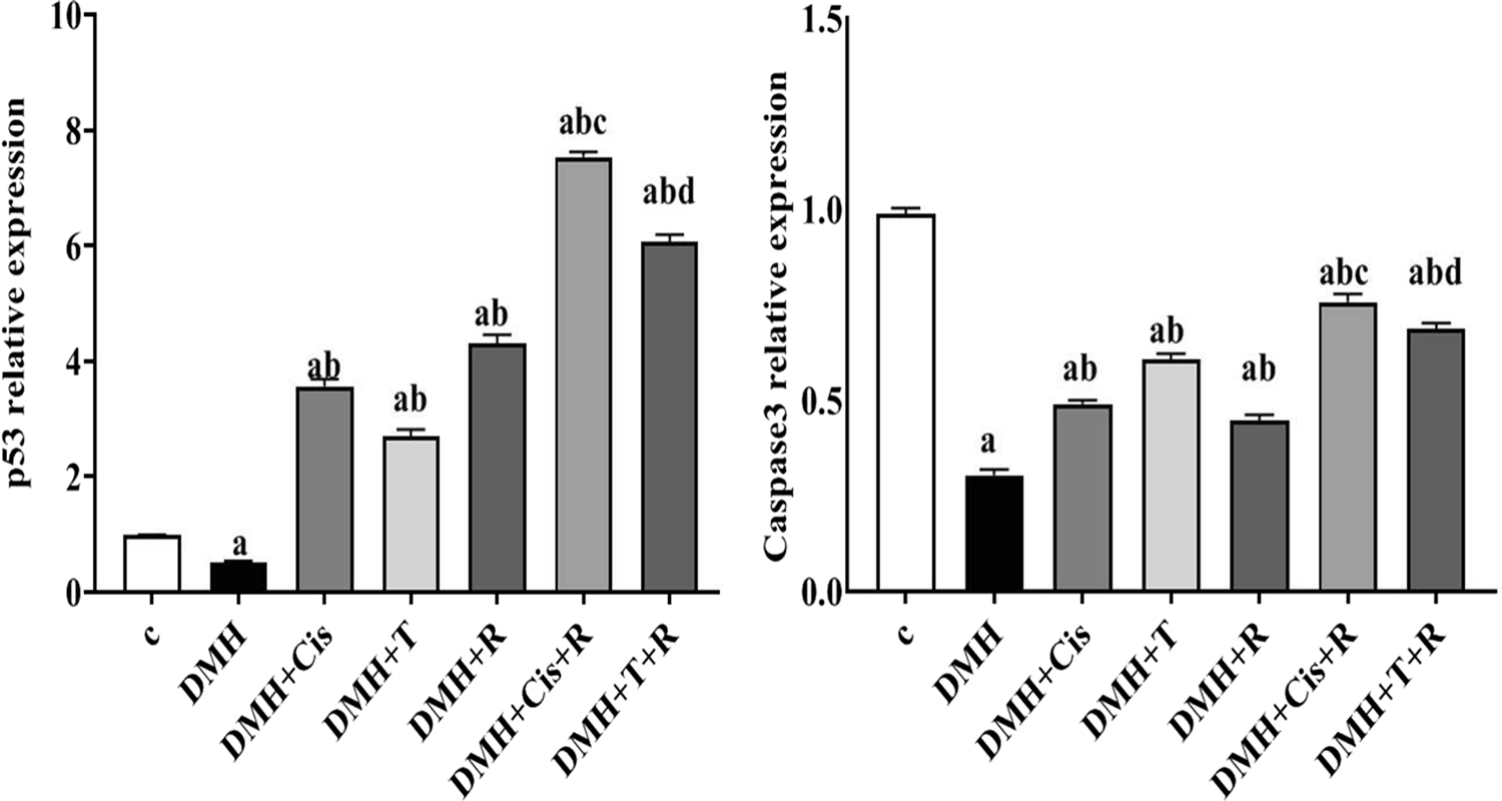
Effect of thiophene derivative and low dose γ-irradiation on *p53* and *caspase-3* expression. C: control, DMH: dimethyl hydrazine, Cis: cisplatin, T: thiophene derivative, R: radiation. Values are the mean ± SEM (n = 6). ^a^Significant change compared to the control group. ^b^Significant change compared to DMH group. ^C^Significant change compared to DMH + Cis. ^d^Significant change compared to DMH + T

RT-PCR analysis showed a pronounced decrease (p < 0.05) in *p53* and *caspase-3* mRNA levels in DMH-treated animals compared to the healthy control group. In the groups treated with DMH + T, DMH + cis, or DMH + R, the expression of *caspase-3* was significantly increased. Moreover, combined treatment with thiophene derivative or cisplatin and low dose γ-irradiation restored the expression of *caspase-3* to levels comparable with those of the normal healthy controls (Fig. 7). Treating the DMH-mice with thiophene derivative, cisplatin, or radiation caused ~ 3–fourfold-induction in the *p53* expression. The combined treatment with thiophene derivative or cisplatin, with radiation resulted in a significant augmentation in the *p53* transcript level with ~ 6–eightfold-induction.

### Effect of thiophene derivative and low dose γ-irradiation on p53 and caspase-3 levels

**Fig. 8:**
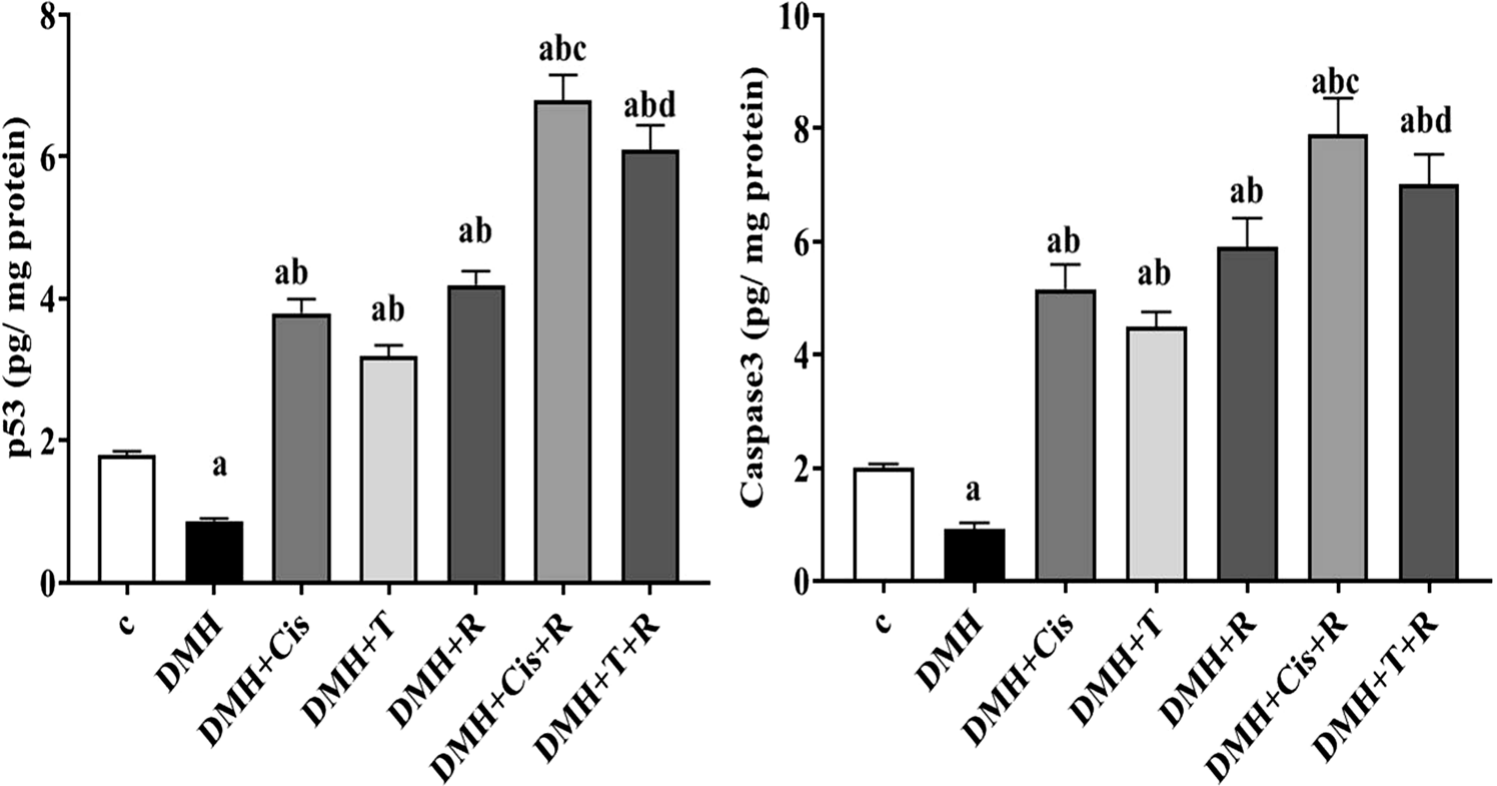
Effect of thiophene derivative and γ-irradiation on the cleaved caspase3 and phosphorylated p53 levels. C: control, DMH: dimethyl hydrazine, Cis: cisplatin, T: thiophene derivative, R: radiation. Values are the mean ± SEM (n = 6). ^a^Significant change compared to the control group. ^b^Significant change compared to DMH group. ^C^Significant change compared to DMH + Cis. ^d^Significant change compared to DMH + T

As compared to the healthy control group, colonic tissue contents of cleaved caspase 3 and phosphorylated p53 were significantly (p < 0.05) decreased in (DMH) group in agreement with the qPCR results. Treatment of DMH-mice with thiophene derivative, cisplatin, or radiation enhanced apoptosis as revealed by the significant elevation in the phosphorylated p53 and cleaved caspase-3 levels by ~ 4–sixfold compared to untreated tumor bearing mice. Exposure of tumor bearing mice to γ-irradiation in combination with thiophene or cisplatin was associated with a sharp further increase (~ 7–eightfold) in the cleaved caspase-3 and phosphorylated p53 levels in tumor tissue (Fig. 8).

### Evaluation of apoptosis by flow cytometry

**Fig. 9:**
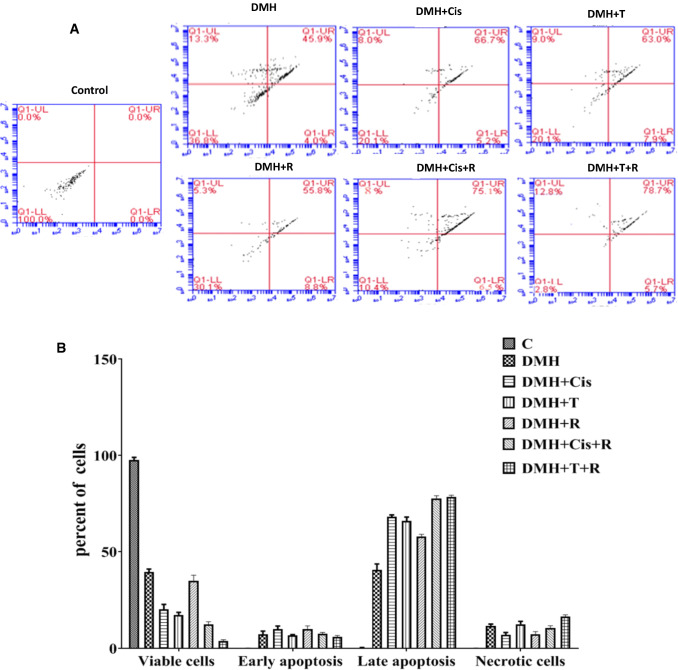
Apoptosis detected by flow cytometry with Annexin V-FITC conjugated with PI staining. A. a representative from each group, B. the means of three samples from each group. C: control, DMH: dimethyl hydrazine, Cis: cisplatin, T: thiophene derivative, R: radiation. Values are the mean ± SEM (n = 3). ^a^Significant change compared to the control group. ^b^Significant change compared to DMH group. ^C^Significant change compared to DMH + Cis. ^d^Significant change compared to DMH + T. The upper right and upper left quadrants represent the late apoptotic and necrotic cells, respectively. The lower left and lower right quadrants represent the viable and early apoptotic cells, respectively in percent of the total gated cells

As can be seen from Fig. 9, apoptosis in the DMH group was significantly decreased compared to all other groups. Figure 9A shows the results of one representative from each group, while Fig. 9B shows the results of means of three samples from each group. The early apoptotic cells% increased from 4% in the DMH group to 8.8, 7.9, and 5.2% in the DMH groups treated with radiation, thiophene derivative, or cisplatin, respectively. Concomitant treatment with cisplatin + radiation elevated that percentage to 6.5%. The late apoptotic cells% increased from 45.9% in the DMH group to 55.8, 63.0, and 66.7% upon treatment with radiation, thiophene derivative, or cisplatin, respectively. Concomitant treatment with thiophene derivative + radiation or cisplatin + radiation elevated the late apoptotic cells percentage to 78.7 and 75.1%, respectively as shown in Fig. 9A. It is noteworthy that the highest percentage of late apoptotic cells and the lowest percentage of viable cells were all reported in the group treated with thiophene derivative + radiation.

### Histopathological findings 

**Fig. 10:**
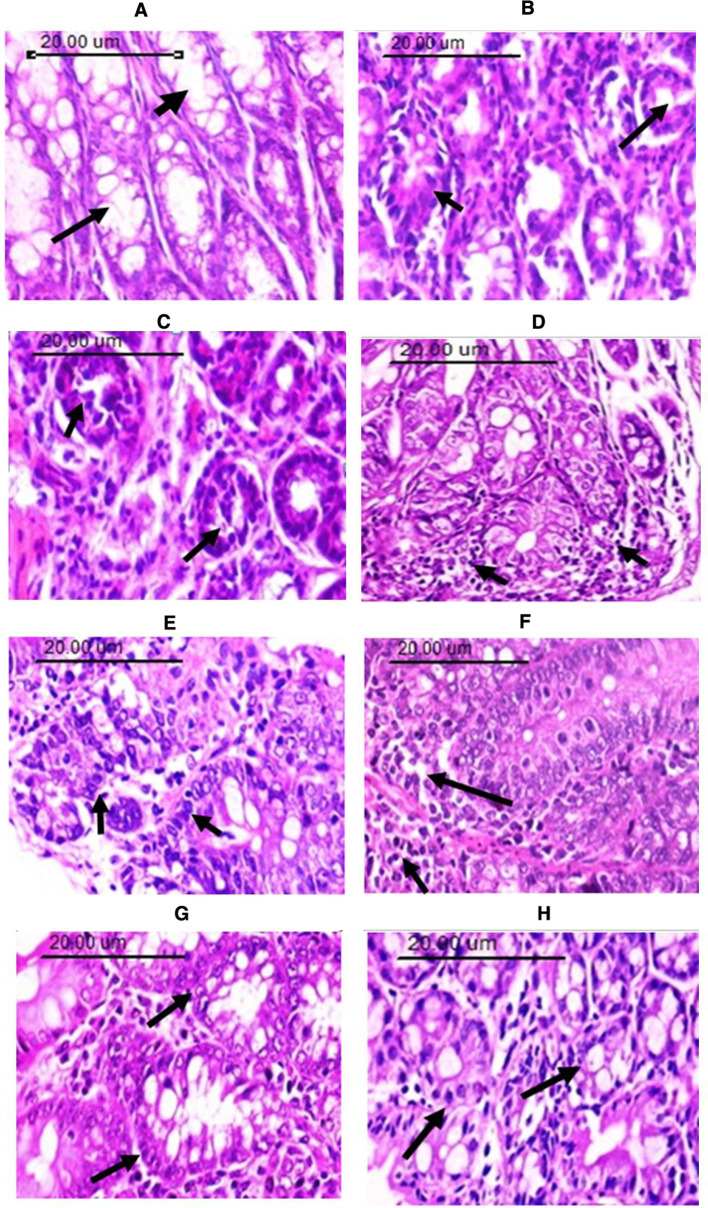
Photomicrographs of mouse colon sections stained with hematoxylin and eosin showing **A** Normal control, the arrow refers to intestinal glands lined by high cuboidal epithelial cells with numerous numbers of goblet cells, **B**, **C** Dysplasia of the mucosal lining epithelium as well as glandular structure with lose of goblet cells formation, pleomorphism in the cells as well as hyperchromatic nuclei of DMH treated animals. The arrow in (B) indicates crowded glands with irregularity in shape and size, and in **C** indicates intestinal glands with elongated nuclei, loss of cell polarity, increased mitotic activity and lack of goblet cells **D** Dysplasia in lining glandular mucosal epithelium with goblet cells formation in the thiophene derivative treated group. The arrow refers to few numbers of goblet cells with decreased number of inflammatory cells **E** Dysplasia of the mucosal lining epithelium with pleomorphic nuclei (arrow) with goblet cells formation in the γ-irradiated group. **F** Massive inflammatory cells permeation in the lamina propria of the mucosa, muscularis mucosa and submucosa (arrow) in colon from cisplatin treated group. **G** Mild degree of dysplastic changes and well differentiated intestinal glands (arrow) in DMH + T + R group. **H** Moderate degree glandular epithelia dysplasia and more crowded glands (arrow) in DMH + cis + R group. (H&E × 200)

The colon of control mice revealed normal histological architecture of intestinal mucosa. The villi lined by a single layer of tall columnar cells with oval basal nuclei and numerous goblet cells dispersed between the columnar cells. The intestinal crypts were regularly arranged and lined by columnar cells. The intestinal glands appeared intact and lined by high cuboidal epithelial cells without any pathological alterations. Inflammatory reaction and hemorrhage were not detected (Fig. 10A). On the other side, mice treated with DMH showed dysplastic changes, aberrant crypt foci (ACF) and intestinal glands with elongated nuclei, loss of cell polarity, increased mitotic activity, absence of goblet cells, and narrow lumens in epithelial cells of aberrant crypt foci. Cell dysplasia with various degrees were seen, with more packed glands that were uneven in shape and size. Complex glands with some areas of solid growth were detected (grade 4). Inflammatory cells, mainly lymphocytes and macrophages, infiltrating more than 20 per medium power field were noticed in lamina propria of the mucosa, muscularis mucosa, and submucosa score 3 (Fig. 10B, C). Colon specimens from tumor bearing-mice treated with thiophene derivative or exposed to γ-radiation showed significant mild and moderate dysplasia and anaplasia with irregular shape and size with some complexity of glands which contained apparent secondary structure (grade 2). Few goblet cells were detected with decreased number of inflammatory cells (10–20) per medium power field score 2 (Fig. 10D, E, respectively). Animals treated with cisplatin showed significant changes from untreated group (DMH). The intestinal glands and ACF showed loss of goblet cells, dysplasia of epithelial lining with pleomorphic nuclei, and reduction of mitotic activity. Some complexity glands contained apparent secondary structure (grade 2). More than 20 inflammatory cells permeated the mucosa's lamina propria, muscularis mucosa, and submucosa (score 3) (Fig. 10F).

In the group treated with DMH + T + R, mucosal glands showed mild hyperplasia, mild degree of dysplastic changes and well differentiated intestinal glands (grade 1). Mild changes of absorptive and goblet cells. The lamina propria of the mucosa, muscularis mucosa, and submucosa was infiltrated with few inflammatory cells (less than 10, score 1) (Fig. 10G). Colon sections from the group treated with DMH + cis + R showed moderate changes of absorptive and goblet cells with moderate degree glandular epithelial dysplasia with more crowded glands and some complexity with glands containing apparent secondary structure grade 2. The lamina propria of the mucosa, muscularis mucosa, and submucosa was infiltrated with very few (less than 10, score 1) inflammatory cells (Fig. 10H).

## Discussion

The high incidence of cancer and the high cost of its treatment are driving the search for new, selective, and effective chemotherapeutic agents. Thiophene-containing molecules have been proven as promising anticancer agents [13]. The earliest identifiable lesions in the progression of colon cancer are ACF. The hypothesis that ACF are truly preneoplastic lesions and that the quantity of ACF is indicative of later tumor growth is backed up by considerable evidence [28]. The presence of a variety of immune cells in the tumor microenvironment shows that inflammation is an important event in multiple features of CRC [29]. Therefore, we evaluated the effects of the thiophene derivative and radiation on the inflammatory markers, COX2 and iNOS in colon tissue. DMH in the current study induced CRC in mice and increased the activities of COX2 and iNOS in colon tissues in accordance with previous studies [4, 30, 31]. COX2 is the rate-limiting enzyme for prostaglandin synthesis and is one of the main players of inflammation, was shown to have a major role in cancer progression, apoptosis, invasion, angiogenesis and metastases [4]. High levels of COX2 have been noticed in about 50% of adenoma and more than 80% of adenocarcinomas [32]. More important, inhibitors of this enzyme significantly reduce the incidence of CRC [33]. Our results revealed that, thiophene derivative or radiation significantly decreased COX2 activity, which could be due to their anti-inflammatory potential. Thiophene derivative along with radiation exposure was more efficient than each of them alone in reducing COX2 activity. In human tumors, a combination of radiotherapy with COX2 inhibitors has been found to boost the therapeutic outcome. COX2 inhibitors can lower inflammatory factors, thereby modulating macrophage recruitment and activation of the anticancer immune milieu; restrict tumor angiogenesis by downregulating vascular endothelial growth factor; and promote tumor cell apoptosis by inhibiting the PI3K/Akt signaling pathway [34]. The probability that such combination can enhance radiosensitivity of tumors could not be ruled out. COX2 inhibitors have been shown in clinical trials to affect the development and progression of cancer. Reduction of COX2 activity is linked to increased tumor radiosensitivity without harming normal tissue in animal models. Preclinical research suggests that the main mechanism is a direct increase in cellular radiation sensitivity and inhibition of tumor neovascularization. [35, 36]. Disubstituted thiophene derivatives were found to inhibit COX2 that is upregulated in many types of cancers. The inhibition of this enzyme is essential for the suppression of tumor development and metastasis [37].

Under normal settings, inducible nitric oxide synthase (iNOS), which produces nitric oxide (NO), is not present in most cells. This depends on the availability of substrate, cofactors, and oxygen tension. However, in many types of cancer, these regulation systems are disrupted resulting in the continuous production and activation of iNOS [38]. iNOS has been shown to play a role in the development of human CRC. iNOS is highly expressed in 60% of human colon adenomas, whereas it is undetectable in normal intestinal tissues [31, 39]. These findings show that iNOS could be used as an early indicator of inflammation and possible subsequent colon cancer. It is well established that iNOS and COX2 are linked to a poor prognosis and high tumor aggressiveness. In colorectal cancer, the proangiogenic action of nitric oxide is mediated by COX2 [40]. In our study, thiophene derivative and gamma radiation significantly attenuated the iNOS activity and again, the combination of thiophene derivative + radiation was more efficient than each treatment alone. Altogether, we conclude that thiophene derivative is an efficient chemotherapy for CRC by modulating iNOS/COX2 system and can sensitize the tumor tissue to radiation. Thus, we confirm that iNOS/COX2 is an attractive biomarker diagnosis and prognosis of CRC.

Cancer is a complex multifactorial disease, therefore, many molecular targets and pathways should be investigated. DMH caused an over-expression of KRAS and a down regulation in PPARγ at the genomic and proteomic levels. Thiophene derivative or radiation ameliorated the changes in the expression of KRAS and PPARγ. Concomitant treatment with thiophene derivative and radiation augmented the elevation in the PPARγ and KRAS expression, but the latter did not achieve a statistical significance. The changes at the genomic levels were reflected by parallel change at the protein levels. ACF has mutations in the adenomatous polyposis coli (APC) and the KRAS oncogenes that promote abnormal cell proliferation [41]. *KRAS* is a commonly mutated oncogene in approximately 40% of all CRC cases. *KRAS* mutations result in constitutive activation of the KRAS protein, which persistently stimulate PI3K-Akt and RAS-RAF-MAPK pathways leading to cell proliferation and survival, and ultimately to tumorigenesis [42, 43]. KRAS also promotes cancer metastasis and increases resistance to chemotherapy in many cancer types including CRC [42, 44].

Peroxisome proliferator activated receptor gamma (PPARγ) have emerged as essential regulators of inflammation. In a variety of malignancies, PPARγ ligands have been found to have an anti-proliferative impact. These ligands have the ability to cause apoptosis through the tumor protein p53 (TP53) pathway [45]. Decreased expression of *PPARγ* has been observed in many tumor types and is usually associated with poor prognosis in cancer patients. COX2 and PPARγ signaling pathways are intertwined**.** PPARγ ligands suppress COX2 expression induced by LPS in macrophages and epithelial cells [46]. The COX2 metabolite 15d-PGJ2 is a PPARγ ligand, and increased 15d-PGJ2 synthesis during inflammation resolution downregulates COX2 via a negative feedback loop involving PPARγ and NF-κB [47].

The major effects seen after DMH were the elevations in the late apoptotic and necrotic cells. This could be attributed to the toxicity of DMH and the continuous genomic modification and genomic instability induced by DMH [48]. There is a growing body of evidence demonstrating a disruption in the balance of cell proliferation and apoptosis during CRC formation. The proliferation exceeds the apoptosis and finally apoptosis is inhibited. Apoptosis failure and inhibition can lead to an imbalance in intestinal epithelial cell homeostasis, which can lead to the development of CRC and a poor response to radiotherapy and/or chemotherapy [49]. The mutation or reduction in the tumor suppressor genes (TP53) and oncogenes (KRAS) will result in an inefficient apoptosis. This was typified in the current study by the reductions in the phosphorylated p53 and cleaved caspase levels after DMH injections at the genomic and proteomic levels. Failure to eliminate altered colonic cells while maintaining a high rate of proliferation is an early indicator of CRC carcinogenesis. The accumulation of continuously modified colonocytes will result in the creation of early adenoma, which will subsequently proceed to adenocarcinoma, and then to late stage CRC [50]. The reason behind the elevation of late apoptosis caused by DMH in the current study is not clear.

The present results revealed that the expression level of p53 and caspase-3 were upregulated after the treatment with thiophene, cisplatin, or radiation. Cisplatin was previously shown to have similar effects [51, 52]. Our study displayed that thiophene derivative when combined with radiation, induced apoptosis which was manifested by ameliorative decrease in KRAS expression associated with a significant increase in PPARγ, cleaved caspase3, and phosphorylated p53 indicating the proapoptotic activity of thiophene derivative, radiation, and their combination. This was also confirmed by Annexin V assay. The "guardian of the genome" is the tumor-suppressor gene TP53, which encodes the protein p53 [53]. In reaction to DNA damage, p53 causes apoptosis or cell cycle arrest. The pro-apoptotic activity of thiophene derivative could be attributed to its ability to cleave the genomic DNA and induce p53 cascade. p53 upregulates pro-apoptotic genes of both extrinsic and intrinsic pathways, and represses the expression of anti-apoptotic genes such as BCL-2 [54, 55]. Kaur et al. showed that another thiophene compound combined with radiation increased apoptosis by Annexin V assay [45]. Ionizing radiations have been shown to cause apoptosis by activating JNK pathways [56]. PPARγ ligands have also been demonstrated to activate MAPK [57], which includes JNK pathway. The phosphorylation of PPAR by JNK increases its transcriptional activity [58]. PTEN, which is implicated in cell cycle arrest and death, is also induced by PPAR [59]. Apoptosis activation has been linked to elevated levels of cleaved PPAR and cleaved caspase-3 [60]. In the present study, the thiophene derivative up-regulated cleaved capase-3 and PPAR proteins in the colon cells.

The histopathological examination of colon from DMH-treated mice revealed many malignant disturbances in the colon architecture such adenomatous polyps dysplastic changes, aberrant crypt foci (ACF), loss of cell polarity, increased mitotic activity, lack of goblet cells, different degrees of cell dysplasia in accordance with previous studies [20, 21, 61]. The administration of DMH-injected mice with thiophene derivative or gamma irradiation improved most of these pathological changes. Similar results for irradiation were previously reported [62]. Cisplatin did not affect the adenomatous hyperplasia but significantly reduced the inflammatory cell infiltration and glandular hyperplasia in accordance with a previous study [63]. The thiophene derivative and gamma irradiation together were much better than CP in ameliorating the pathology of colorectal tumors.

## Conclusions

The goal of the present study was to investigate the antitumor efficacy of a new thiophene derivative against CRC in mice and explore the possible associated molecular pathways. The potential of this thiophene to sensitize the CRC tumor tissue to a low dose of gamma irradiation was also investigated. Thiophene derivative and radiation reduced the mortality, tumor incidence and tumor multiplicity caused by DMH. The most noteworthy finding of the current finding is that thiophene derivative induced down regulation of inflammation (COX2 and iNOS) and cell survival pathway proteins (KRAS) contributing to a negative regulation of cell cycle regulatory proteins, which resulted in cell cycle arrest, and apoptosis (elevated PPAR, p53, and caspase 3). Our data highlighted the potential of this thiophene derivative to sensitize the CRC to the ionizing radiation. Taken together, this new thiophene derivative is a promising therapeutic candidate for treatment of colorectal cancer either alone or in combination with a low dose of gamma irradiation.

## Data Availability

All data presented in this manuscript are reported in the manuscript.

## References

[1] Sung H, Ferlay J, Siegel RL, Laversanne M, Soerjomataram I, Jemal A, Bray F (2021). Global cancer statistics 2020: GLOBOCAN estimates of incidence and mortality worldwide for 36 cancers in 185 countries. CA Cancer J Clin.

[2] Fernández J, Silván B, Entrialgo-Cadierno R, Villar CJ, Capasso R, Uranga JA, Lombó F, Abalo R (2021). Antiproliferative and palliative activity of flavonoids in colorectal cancer. Biomed Pharmacother.

[3] Bruce WR, Corpet DE (1996). The colonic protein fermentation and insulin resistance hypotheses for colon cancer etiology: experimental tests using precursor lesions. Eur J Cancer Prev.

[4] Ghazizadeh Darband S, Saboory E, Sadighparvar S, Kaviani M, Mobaraki K, Jabbari N, Majidinia M (2020). The modulatory effects of exercise on the inflammatory and apoptotic markers in rats with 1,2-dimethylhydrazine-induced colorectal cancer. Can J Physiol Pharmacol.

[5] Hamiza OO, Rehman MU, Tahir M, Khan R, Khan AQ, Lateef A, Ali F, Sultana S (2012). Amelioration of 1,2 dimethylhydrazine (DMH) induced colon oxidative stress, inflammation and tumor promotion response by tannic acid in Wistar rats. Asian Pac J Cancer Prev.

[6] An N, Sun Y, Ma L, Shi S, Zheng X, Feng W, Shan Z, Han Y, Zhao L, Wu H (2020). Helveticoside exhibited p53-dependent anticancer activity against colorectal cancer. Arch Med Res.

[7] Campbell PI, Al-Nasser IA (1996). Renal insufficiency induced by cisplatin in rats is ameliorated by cyclosporin A. Toxicology..

[8] Hasan HF, Rashed LA, El Bakary NM (2021). Concerted outcome of metformin and low dose of radiation in modulation of cisplatin induced uremic encephalopathy via renal and neural preservation. Life Sci.

[9] Ali BH, Al Moundhri MS (2006). Agents ameliorating or augmenting the nephrotoxicity of cisplatin and other platinum compounds: a review of some recent research. Food Chem Toxicol.

[10] El-Sayed WM, Hussin WA (2013). Antimutagenic and antioxidant activity of novel 4-substituted phenyl-2,2'-bichalcophenes and aza-analogs. Drug Des Devel Ther.

[11] Hussin WA, Ismail MA, Alzahrani AM, El-Sayed WM (2014). Evaluation of the biological activity of novel monocationic fluoroaryl-2,2'-bichalcophenes and their analogues. Drug Des Devel Ther.

[12] Ismail MA, Arafa RK, Youssef MM, El-Sayed WM (2014). Anticancer, antioxidant activities, and DNA affinity of novel monocationic bithiophenes and analogues. Drug Des Devel Ther.

[13] Ismail MA, Youssef MM, Arafa RK, Al-Shihry SS, El-Sayed WM (2017). Synthesis and antiproliferative activity of monocationic arylthiophene derivatives. Eur J med chem.

[14] Ismail MA, Negm A, Arafa RK, Abdel-Latif E, El-Sayed WM (2019). Anticancer activity, dual prooxidant/antioxidant effect and apoptosis induction profile of new bichalcophene-5-carboxamidines. Eur J Med Chem.

[15] Fitzgerald DJ, Anderson JN (1999). Selective nucleosome disruption by drugs that bind in the minor groove of DNA. J Biol Chem.

[16] Nhili R, Peixoto P, Depauw S, Flajollet S, Dezitter X, Munde MM, Ismail MA, Kumar A, Farahat AA, Stephens CE, Duterque-Coquillaud M, David Wilson W, Boykin DW, David-Cordonnier MH (2013). Targeting the DNA-binding activity of the human ERG transcription factor using new heterocyclic dithiophene diamidines. Nucleic Acids Res.

[17] Calabrese EJ, Baldwin LA (2001). Scientific foundations of hormesis. Crit Rev Toxicol.

[18] Feinendegen LE (2005). Evidence for beneficial low-level radiation effects and radiation hormesis. Br J Radiol.

[19] El Bakary NM, Azab KhSh, Hanafy SM, Abdul Aziz GM, Fayed AM (2021). Calcitriol revises aromatase gene expression in ehrlich solid tumor bearing mice exposed to low dose gamma radiation. JCRCT.

[20] Abdel-Rasol M, El-Beih NM, Yahya SMM, Ismail MA, El-Sayed WM (2020). The antitumor activity of a novel fluorobenzamidine against dimethylhydrazine- induced colorectal cancer in rats. Anticancer Agents Med Chem.

[21] Abdel-Rasol M, El-Beih N, Yahya S, El-Sayed W (2022). The antitumor activity of ginger against colorectal cancer induced by dimethylhydrazine in rats. Anticancer Agents Med Chem.

[22] Tanaka T, Kohno H, Suzuki R, Yamada Y, Sugie S, Mori H (2003). A novel inflammation-related mouse colon carcinogenesis model induced by azoxymethane and dextran sodium sulfate. Cancer Sci.

[23] Thierry AR, Mouliere F, Gongora C, Ollier J, Robert B, Ychou M, Del Rio M, Molina F (2010). Origin and quantification of circulating DNA in mice with human colorectal cancer xenografts. Nucleic Acids Res.

[24] Abdelrahman IY, Helwa R, Elkashef H, Hassan NH (2015). Induction of P3NS1 myeloma cell death and cell cycle arrest by simvastatin and/or γ-radiation. Asian Pac J Cancer Prev.

[25] Mingone CJ, Gupte SA, Quan S, Abraham NG, Wolin MS (2003). Influence of heme and heme oxygenase-1 transfection of pulmonary microvascular endothelium on oxidant generation and cGMP. Exp Biol Med (Maywood).

[26] Bancroft JD, Stevens A, Turner DR (2013). Theory and practice of histological techniques.

[27] Shia J, Schultz N, Kuk D, Vakiani E, Middha S, Segal NH, Hechtman JF, Berger MF, Stadler ZK, Weiser MR, Wolchok JD, Boland CR, Gönen M, Klimstra DS (2017). Morphological characterization of colorectal cancers in the cancer genome atlas reveals distinct morphology-molecular associations: clinical and biological implications. Mod Pathol.

[28] Mori M, Hata K, Yamada Y, Kuno T, Hara A (2005). Significance and role of early-lesions in experimental colorectal carcinogenesis. Chem Biolo Interact.

[29] Majidinia M, Alizadeh E, Yousefi B, Akbarzadeh M, Mihanfar A, Rahmati-Yamchi M, Zarghami N (2017). Co-inhibition of notch and NF-κB signaling pathway decreases proliferation through downregulating IκB-α and Hes-1 expression in human ovarian cancer OVCAR-3 cells. Drug Res (Stuttg).

[30] Arber N, Eagle CJ, Spicak J, Rácz I, Dite P, Hajer J, Zavoral M, Lechuga MJ, Gerletti P, Tang J, Rosenstein RB, Macdonald K, Bhadra P, Fowler R, Wittes J, Zauber AG, Solomon SD, Levin B (2006). PreSAP trial investigators. Celecoxib for the prevention of colorectal adenomatous polyps. N Engl J Med.

[31] Wang H, Wang L, Xie Z (2020). Nitric oxide (NO) and NO synthases (NOS)-based targeted therapy for colon cancer. Cancers (Basel).

[32] Eberhart CE, Coffey RJ, Radhika A, Giardiello FM, Ferrenbach S, DuBois RN (1994). Up-regulation of cyclooxygenase 2 gene expression in human colorectal adenomas and adenocarcinomas. Gastroenterology.

[33] Sheehan KM, Sheahan K, O'Donoghue DP, MacSweeney F, Conroy RM, Fitzgerald DJ, Murray FE (1999). The relationship between cyclooxygenase-2 expression and colorectal cancer. JAMA.

[34] Li S, Jiang M, Wang L, Yu S (2020). Combined chemotherapy with cyclooxygenase-2 (COX-2) inhibitors in treating human cancers: Recent advancement. Biomed Pharmacother.

[35] Choy H, Milas L (2003). Enhancing radiotherapy with cyclooxygenase-2 enzyme inhibitors: a rational advance?. J Natl Cancer Inst.

[36] Cheki M, Yahyapour R, Farhood B, Rezaeyan A, Shabeeb D, Amini P, Rezapoor S, Najafi M (2018). COX-2 in radiotherapy: a potential target for radioprotection and radiosensitization. Curr Mol Pharmacol.

[37] Rakesh KS, Jagadish S, Swaroop TR, Mohan CD, Ashwini N, Harsha KB, Zameer F, Girish KS, Rangappa KS (2015). Anti-cancer activity of 2,4-disubstituted thiophene derivatives: dual inhibitors of lipoxygenase and cyclooxygenase. Med Chem.

[38] Kielbik M, Szulc-Kielbik I, Klink M (2019). The potential role of iNOS in ovarian cancer progression and chemoresistance. Int J Mol Sci.

[39] Vannini F, Kashfi K, Nath N (2015). The dual role of iNOS in cancer. Redox Biol.

[40] Cianchi F, Cortesini C, Fantappiè O, Messerini L, Sardi I, Lasagna N, Perna F, Fabbroni V, Di Felice A, Perigli G, Mazzanti R, Masini E (2004). Cyclooxygenase-2 activation mediates the proangiogenic effect of nitric oxide in colorectal cancer. Clin Cancer Res.

[41] Smith AJ, Stern HS, Penner M, Hay K, Mitri A, Bapat BV, Gal-linger S (1994). Somatic APC and K-ras codon 12 mutations in aberrant crypt foci from human colons. Cancer Res.

[42] Meng M, Zhong K, Jiang T, Liu Z, Kwan HY, Su T (2021). The current understanding on the impact of KRAS on colorectal cancer. Biomed Pharmacother.

[43] Zhu G, Pei L, Xia H (2021). Role of oncogenic KRAS in the prognosis, diagnosis and treatment of colorectal cancer. Mol Cancer.

[44] Shingu T, Holmes L, Henry V, Wang Q, Latha K, Gururaj AE, Gibson LA, Doucette T, Lang FF, Rao G, Yuan L, Sulman EP, Farrell NP, Priebe W, Hess KR, Wang YA, Hu J, Bögler O (2016). Suppression of RAF/MEK or PI3K synergizes cytotoxicity of receptor tyrosine kinase inhibitors in glioma tumor-initiating cells. J Transl Med.

[45] Kaur S, Nag A, Gangenahalli G, Sharma K (2019). Peroxisome proliferator activated receptor gamma sensitizes non-small cell lung carcinoma to gamma irradiation induced apoptosis. Front Genet.

[46] Subbaramaiah K, Lin DT, Hart JC, Dannenberg AJ (2001). Peroxisome proliferator-activated receptor gamma ligands suppress the transcriptional activation of cyclooxygenase-2. Evidence for involvement of activator protein-1 and CREB-binding protein/p300. J Biol Chem.

[47] Hazra S, Peebles KA, Sharma S, Mao JT, Dubinett SM (2008). The role of PPARgamma in the cyclooxygenase pathway in lung cancer. PPAR Res.

[48] Ansari B, Coates PJ, Greenstein BD, Hall PA (1993). In situ end-labelling detects DNA strand breaks in apoptosis and other physiological and pathological states. J Pathol.

[49] Watson AJ (1995). Review article: manipulation of cell death–the development of novel strategies for the treatment of gastrointestinal disease. Aliment Pharmacol Ther.

[50] Ismail NI, Othman I, Abas F, Lajis NH, Naidu R (2019). Mechanism of apoptosis induced by curcumin in colorectal cancer. Int J Mol Sci.

[51] Peng ZH, Xing TH, Qiu GQ, Tang HM (2001). Relationship between Fas/FasL expression and apoptosis of colon adenocarcinoma cell lines. World J Gastroenterol.

[52] Vondálová Blanárová O, Jelínková I, Szöor A, Skender B, Soucek K, Horváth V, Vaculová A, Andera L, Sova P, Szöllosi J, Hofmanová J, Vereb G, Kozubík A (2011). Cisplatin and a potent platinum(IV) complex-mediated enhancement of TRAIL-induced cancer cells killing is associated with modulation of upstream events in the extrinsic apoptotic pathway. Carcinogenesis.

[53] Altieri DC (2008). New wirings in the survivin networks. Oncogene.

[54] Hotchkiss RS, Strasser A, McDunn JE, Swanson PE (2009). Cell death. N Engl J Med.

[55] Ghatage DD, Gosavi SR, Ganvir SM, Hazarey VK (2012). Apoptosis: molecular mechanism. J Orofac Sci.

[56] Dent P, Yacoub A, Contessa J, Caron R, Amorino G, Valerie K, Hagan MP, Grant S, Schmidt-Ullrich R (2003). Stress and radiation-induced activation of multiple intracellular signaling pathways. Radiat Res.

[57] Gardner OS, Dewar BJ, Graves LM (2005). Activation of mitogen-activated protein kinases by peroxisome proliferator-activated receptor ligands: an example of nongenomic signaling. Mol Pharmacol.

[58] Yin R, Dong YG, Li HL (2006). PPARgamma phosphorylation mediated by JNK MAPK: a potential role in macrophage-derived foam cell formation. Acta Pharmacol Sin.

[59] Farrow B, Evers BM (2003). Activation of PPARgamma increases PTEN expression in pancreatic cancer cells. Biochem Biophys Res Commun.

[60] Boulares AH, Yakovlev AG, Ivanova V, Stoica BA, Wang G, Iyer S, Smulson M (1999). Role of poly(ADP-ribose) polymerase (PARP) cleavage in apoptosis. Caspase 3-resistant PARP mutant increases rates of apoptosis in transfected cells. J Biol Chem.

[61] Zaafar DK, Zaitone SA, Moustafa YM (2014). Role of metformin in suppressing 1,2-dimethylhydrazine-induced colon cancer in diabetic and non-diabetic mice: effect on tumor angiogenesis and cell proliferation. PLoS ONE.

[62] Zahran WE, Elsonbaty SM, Moawed FSM (2017). Lactobacillus rhamnosus ATCC 7469 exopolysaccharides synergizes with low level ionizing radiation to modulate signaling molecular targets in colorectal carcinogenesis in rats. Biomed Pharmacother.

[63] Li QC, Liang Y, Hu GR, Tian Y (2016). Enhanced therapeutic efficacy and amelioration of cisplatin-induced nephrotoxicity by quercetin in 1,2-dimethyl hydrazine-induced colon cancer in rats. Indian J Pharmacol.

